# Olfactory perception and blindness: a systematic review and meta-analysis

**DOI:** 10.1007/s00426-018-1035-2

**Published:** 2018-06-12

**Authors:** Agnieszka Sorokowska, Piotr Sorokowski, Maciej Karwowski, Maria Larsson, Thomas Hummel

**Affiliations:** 1 Smell and Taste Clinic, Department of Otorhinolaryngology, TU Dresden, Fetscherstr. 74, 01307 Dresden, Germany; 2 Institute of Psychology, University of Wroclaw, pl. Dawida 1, 50-527 Wrocław, Poland; 3 Gösta Ekman Laboratory, Department of Psychology, Stockholm University, Frescati Hagväg 9A, 10691 Stockholm, Sweden

## Abstract

Anecdotal reports suggest that blind people might develop supra-normal olfactory abilities. However, scientific evidence shows a mixed pattern of findings. Inconsistent observations are reported for both sensory-driven olfactory tasks (e.g., odor threshold) and higher-order olfactory functions (e.g., odor identification). To quantify the evidence systematically, we conducted a review and meta-analysis. Studies were included if they examined olfactory function (i.e., odor threshold, odor discrimination, free odor identification, or cued odor identification) in blind compared with a sighted control group. Articles were identified through computerized literature search. A total of 18 studies focused on olfactory threshold (*n* = 1227: 590 blind and 637 sighted individuals), 14 studies targeted discrimination (*n* = 940: 455 blind and 485 sighted), 14 studies measured cued identification (*n* = 968: 468 blind and 500 sighted), and 7 studies (*n* = 443: 224 blind and 219 sighted individuals) assessed free identification. Overall, there were no differences in effect sizes between the blind and sighted individuals after correcting the results for publication bias. We additionally conducted an exploratory analysis targeting the role played by three moderators of interests: participants’ age, the proportion of women versus men in each of the studies included into meta-analysis and onset of blindness (early blind vs. late-blind). However, none of the moderators affected the observed results. To conclude, blindness seems not to affect cued/free odor identification, odor discrimination or odor thresholds.

**Electronic supplementary material:**

The online version of this article (10.1007/s00426-018-1035-2) contains supplementary material, which is available to authorized users.

## Introduction

Anecdotal reports suggest that blind people might develop supra-normal olfactory abilities. For example, James Mitchell, a congenitally deaf and blind boy, was allegedly able to follow the odor trail of a person for several miles (Stewart, 1815). Researchers hypothesize that such abilities could result from sensory compensation, i.e., enhanced sensitivity of functioning modalities resulting from deprivation in one or more senses (Kupers & Ptito, 2014). This compensation could emerge due to both central and peripheral reasons. For instance, in sighted individuals, the occipital cortex is activated in visual processing, and recent studies showed occipital activation in blind participants during odor-processing tasks, like odor detection, categorization and discrimination (Kupers et al., 2011; Renier et al., 2013). This suggest that “visual” brain regions could, as a result of functional and structural reorganization, support the processing of olfactory information in the visually impaired (Araneda, Renier, Rombaux, Cuevas, & De Volder, 2016; Kupers & Ptito, 2014). Further, an extensive use and attention to olfactory information in everyday life might promote the development of enhanced olfactory abilities (Cuevas, Plaza, Rombaux, De Volder, & Renier, 2009; Gagnon, Ismaili, Ptito, & Kupers, 2015). This possibility is further confirmed by studies showing a high subjective value of the sense of smell among blind individuals (Beaulieu-Lefebvre, Schneider, Kupers, & Ptito, 2011; Ferdenzi, Coureaud, Camos, & Schaal, 2010). A recent review on the neurobiological aspects of sensory compensation (Araneda et al., 2016) proposed that such training may have a direct effect on olfactory bulb (OB) volume, that in turn influence olfactory function. Indeed, OB volume has been shown to be larger among the early blind individuals as compared to sighted controls (Rombaux et al., 2010). Plasticity in the OB may underlie the enhanced olfactory perception in sighted (Buschhüter et al., 2008; Hummel et al., 2011; Hummel, Haehner, Hummel, Croy, & Iannilli, 2013; Mueller et al., 2005) and among the blind (but see Mazal, Haehner, & Hummel, 2016).

Despite many hypotheses on the etiology of increased olfactory abilities of the blind people, the behavioral studies provide a mixed pattern of findings. As discussed in the following sections, inconsistent observations are reported for both sensory-driven olfactory tasks (e.g., odor detection threshold—see “Olfactory threshold”) and higher-order olfactory functions (e.g., odor identification abilities—see “Olfactory identification”). Although some studies presented below report enhanced olfactory abilities in blindness, there are also observations showing no reliable differences in smell function between blind and sighted individuals. Some degree of heterogeneity of findings may be expected as previous studies had small sample sizes, used different methods, or sampled from different populations (e.g., age, onset of blindness, study site location). However, to determine if there is an actual heterogeneity among the existing observations that is not simply due to chance, we conducted a meta-analysis of available studies targeting olfactory function in blind and sighted individuals.

In the current meta-analysis, we provide a comprehensive examination of olfactory function including odor threshold, odor discrimination, cued odor identification, and free odor identification in blind people as compared with sighted controls. Further, we investigated the potentially moderating roles of age and onset of blindness upon the observed olfactory differences between blind and sighted controls. Below follows a systematic review of the available scientific evidence followed by the systematic quantification of the observations through meta-analytical procedures (for a summary of standardized testing methods see Supplementary File S1).

### Olfactory threshold

Olfactory threshold can be defined as the lowest concentration at which the presence of an odorant is reliably detected (Stevens, 1961). The term “olfactory detection threshold” refers to the ability to detect odorants; it is often referred to as “overall smell sensitivity”. As compared with higher-order olfactory tasks (e.g., odor identification) measurement of odor thresholds pose few demands on cognitive function (Hedner, Larsson, Arnold, Zucco, & Hummel, 2010; Sorokowska, Sorokowski, Hummel, & Huanca, 2013) and appears to draw more on peripheral functions of the olfactory system (e.g., Whitcroft, Cuevas, Haehner, & Hummel, 2016).

Historically, olfactory thresholds were the first smell function targeted in scientific studies on smell in blindness. Griesbach (1899), Cherubino and Salis (1957), and Boccuzzi (1962) found no performance differences in blind and sighted individuals across a range of custom-made olfactory threshold tests. Corroborating these findings, more recent work has reported comparable odor sensitivity in blind and sighted individuals. Here, the Sniffin’ Sticks Test (SST; Hummel, Barz, Pauli, & Kobal, 1998; Hummel, Kobal, Gudziol, & Mackay-Sim, 2007) has been the most commonly used test (Cornell Kärnekull, Arshamian, Nilsson, & Larsson, 2016; Guducu, Oniz, Ikiz, & Ozgoren, 2016; Hamáková, 2008; Luers et al., 2014; Oniz, Erdogan, Bayazit, & Ozgoren, 2011; Schwenn, Hundorf, Moll, Pitz, & Mann, 2002; Sorokowska, 2016). Additionally, researchers using a single staircase phenyl ethyl alcohol (PEA) test (Smith, Doty, Burlingame, & McKeown, 1993), Munich Olfaction Test (MOT; Diekmann, Walger, & von Wedel, 1994; Kruggel, 1989), and air-blast olfactometric method with mint oil and fresh coffee (Zielke & Gawęcki, 2003) observed no differences between sighted and blind individuals. Two studies conducted among blind and sighted children using *n*-butyl alcohol (12-item threshold test; Wakefield, Homewood, & Taylor, 2004, and 13-bottle dilution series; Rosenbluth, Grossman, & Kaitz, 2000) showed no effects of visual impairment on sensory abilities. However, other studies using the SST threshold subtest have demonstrated a superior olfactory performance in blind (Beaulieu-Lefebvre et al., 2011; Çomoğlu et al., 2015; Cuevas et al., 2010). At odds with these observations, Murphy and Cain (1986) who used a *n*-butanol threshold test reported higher olfactory sensitivity in sighted relative to the blind.

### Olfactory discrimination

Assessment of olfactory discrimination ability is often based on non-verbal tasks (Frijters, Kooistra, & Vereijken, 1980; Potter & Butters, 1980) where subjects are confronted with a pair or three odors where they have to find out whether the two odors are different or which of the three odors is different. However, because of many different possibilities in execution of the test results from two odor discrimination tests may not significantly correlate with each other (Weierstall & Pause, 2012). Odor discrimination abilities (as measured with the Sniffin’ Sticks test) have been shown to be associated with executive functioning (Hedner et al., 2010; Sorokowska, Sorokowski, & Hummel, 2014).

Olfactory discrimination tasks were also applied in early works on sensory compensation. By means of a discrimination task involving presentation of 2 odorants in 20 different concentrations, Mahner (1909) concluded that congenitally blind discriminated between odors better than sighted individuals. The effect of blindness on olfactory discrimination has also been tested with a number of different methods. In the group of studies showing comparable performance of the blind and the sighted, researchers used SST (Beaulieu-Lefebvre et al., 2011; Cornell Kärnekull et al., 2016; Guducu et al., 2016; Luers et al., 2014; Majchrzak & Eberhard, 2014; Majchrzak, Eberhard, Kalaus, & Wagner, 2017; Oniz et al., 2011; Schwenn et al., 2002; Sorokowska, 2016), and the Munich Olfaction Test (MOT designed by Kruggel, 1989; this method was used by Diekmann et al., 1994). However, results of studies employing SST were not consistent—some of these studies showed superior discrimination skills of blind people (Çomoğlu et al., 2015; Cuevas et al., 2010), similar to works using a custom set of 30 odorants (Cuevas et al., 2009; Renier et al., 2013; Rombaux et al., 2010). Again, one study demonstrated better olfactory performance in sighted than legally blind subjects in a 16-item discrimination test (Smith et al., 1993).

### Olfactory identification

Measurement of odor identification ability is the most commonly used test of olfactory function in various scientific studies. There are numerous versions of tests available, while the Sniffin’ Sticks (Hummel, Sekinger, Wolf, Pauli, & Kobal, 1997) and the University of Pennsylvania Smell Identification Test (UPSIT; Doty, Shaman, & Dann, 1984; Doty, Shaman, Kimmelman, & Dann, 1984) are most frequently used. Identification may be assessed in an uncued task where no retrieval support is provided (free identification) or by cued identification where a number of alternatives is provided of which one is the target name. Proficiency in odor identification is associated with verbal abilities (Larsson, Nilsson, Olofsson, & Nordin, 2004) and cultural context such that tests need to be specifically adapted for various countries and cultures (e.g., Oleszkiewicz et al., 2016). Although relatively straightforward, minor manipulations in test administration may change test results significantly (e.g., reading the options prior to smelling in a cued odor identification task might significantly decrease performance in this test, Sorokowska, Albrecht, & Hummel, 2015).

Interestingly, no studies have reported superiority of the blind individuals in cued identification. This has been shown with the use of various standardized tests, e.g., the SST (Beaulieu-Lefebvre et al., 2011; Çomoğlu et al., 2015; Cuevas et al., 2010; Guducu et al., 2016; Hamáková, 2008; Luers et al., 2014; Majchrzak & Eberhard, 2014; Majchrzak et al., 2017; Oniz et al., 2011; Schwenn et al., 2002; Sorokowska, 2016; Sorokowska & Karwowski, 2017), UPSIT (Smith et al., 1993), Munich Olfaction Test (MOT, Kruggel, 1989) in Diekmann et al. (1994), Monex 40 Sniffin’ Sticks battery (Freiherr et al., 2012) in a study by Iversen, Ptito, Møller and Kupers (2015). Null effect of blindness was also reported in studies using a variety of custom-made tools, like a set of 30 odors (Cuevas et al., 2009), a set of 38 odorants (Gagnon et al., 2015), and by a test comprising 25 common items identified by blind children (Rosenbluth et al., 2000).

In contrast, a different pattern of findings is obtained for free identification. Although two studies observed comparable performance in free identification using the SST identification test (Sorokowska, 2016; Sorokowska & Karwowski, 2017), others report superior performance among blind compared to sighted. As is true for the other assessed olfactory domains, these methodologies used varied techniques including a set of 30 odorants (Cuevas et al., 2009; Renier et al., 2013; Rombaux et al., 2010), 80 everyday substances (Murphy & Cain, 1986), and 12 odors taken from SST battery (Cornell Kärnekull et al., 2016). Free identification abilities were found to be superior also among blind children; here, researchers applied 16 (Wakefield et al., 2004), or 25 common odors (Rosenbluth et al., 2000).

### Other olfactory abilities

Olfactory abilities encompass various skills, not only these tested by typical smell tests. Such abilities were also compared between blind and sighted participants. Again, some studies demonstrated comparable performance of blind and sighted participants—for example, in retronasal identification test, i.e., in a task involving identification of flavors delivered through participant’s mouth. Null effect of blindness was shown for a test consisting of 38 odorants (Gagnon et al., 2015) and retronasal smell test designed by Heilmann, Strehle, Rosenheim, Damm and Hummel (2002) in the study of Cuevas et al. (2010). Also episodic odor recognition performance (Cornell Kärnekull et al., 2016; Sorokowska & Karwowski, 2017) was similar among blind and sighted individuals. Finally, event-related potentials (ERPs) were analyzed in response to both olfactory and trigeminal stimuli. Trigeminal nerve cells respond to tactile, thermal, or nociceptive stimulation, and trigeminal sensations include stinging, burning, tickling etc. (Hummel & Livermore, 2002; Kleemann et al., 2009). Observed ERPs pattern did not differentiate blind and sighted subjects for neither olfactory nor trigeminal stimuli (Cuevas et al., 2011; Guducu et al., 2016; Schwenn et al., 2002).

Other studies showed that olfactory abilities of blind people were better than those of sighted individuals. The visual deprivation effect was observed, for example, in an odor categorization task involving a set of 30 odorants (Cuevas et al., 2009; Renier et al., 2013) and for free identification time for 38 odorants (Gagnon et al., 2015), or 25 common items tested among blind children (Rosenbluth et al., 2000). The two existing questionnaire studies demonstrated higher olfactory awareness of blind adults (Beaulieu-Lefebvre et al., 2011) and more olfactory-related behaviors (self-assessed reactions to odors in different situations) among blind children compared to their sighted peers (Ferdenzi et al., 2010).

Interestingly, all magnetic resonance studies discussed in the current review have reported olfactory-related superiority in blind individuals. First, they were found to have higher OB volumetric measurements assessed by an MRI scan (Rombaux et al., 2010). Further, fMRI activation patterns differed between the blind and the sighted participants. Researchers observed stronger occipital activation in blind subjects during odor-processing tasks (discrimination or categorization of fruit and flower odors in Renier et al., 2013, and odor detection in Kupers et al., 2011) and stronger response to olfactory stimuli in primary (right amygdala) and secondary (right orbitofrontal cortex and bilateral hippocampus) olfactory areas (Kupers et al., 2011). Finally, in the only existing study on a social aspect of olfaction, i.e., identification of fear from samples of male odor (Iversen et al., 2015), blind people performed better than the sighted.

## Method

### Search strategies

We conducted an extensive literature search to identify empirical studies that involved an evaluation of olfactory sensitivity of the blind people. First, we reviewed articles and research papers in English, Polish, German, Spanish, Italian, and Czech (languages spoken by the authors). The search was performed between July 2016 and May 2018. We searched Google, Google Scholar, Web of Science, DOAJ, EBSCO, PsycExtra, Academic Search Complete, Medline, Health Source: Nursing/Academic Edition, MasterFILE Premier, PsycInfo, PsycArticles, and ERIC databases and used the resources of Elsevier, JSTOR, Science Direct, SAGE Journals, Springer, Taylor & Francis, Wiley, and ProQuest using the following keywords and their combinations: blind*, smell*, olfact*, visual*+impair*. Additionally, we reviewed all works cited in and by the retrieved articles. When a full version of an article was unavailable, we emailed the authors or we tried to localize it in university libraries in the country of origin of the authors. Additionally, when the necessary statistics were incomplete in the full version of an article, we emailed the authors for provision of the data. The studies found are reviewed above and presented in Table 1.

**Table 1:** All studies on olfactory abilities of blind people available in researched sources

	Study	Type of smell test	Included in the meta-analysis	Sample size—blind people			Sample size—sighted people	*g*	Var-*g*	Comments/reasons for exclusion
				Total (proportion of women)	Early blind	Late-blind				
1	Griesbach (1899)	Threshold	Yes	10 (0.00)	6	2	22	− 0.45	0.15	The author reported values for two nostrils separately. We included a lower threshold value to the analysis. Blindness onset for two subjects was not reported
2	Mahner (1909)	Discrimination	Yes	4 (0.00)			4	2.29	0.83	We analyze the average value of hits across two measurements
3	Marcovigi-Bertolini (1942)	No data	No	20			No data			No access to the paper (also in main Italian university libraries)
4	Cherubino and Salis (1957)	Threshold	No	6			6			Lack of necessary data in the paper, no contact with the author
5	Boccuzzi (1962)	Threshold	Yes	100 (0.39)	36	64	100	0.41	0.02	The data for blind subject number 91 could not be used due to a missing value in vanillin threshold measurement
6	Murphy and Cain (1986)	Free identification	Yes	20 (0.65)			20	0.99	0.11	Analysis of the first session out of 3 with the same participants
		Threshold	Yes	18			18	− 1.04	0.13	Two blind subjects and corresponding sighted people were not tested for threshold
7	Smith et al. (1993)	Cued identification	Yes	52 (0.59)			61	− 0.16	0.04	
		Threshold	Yes	39			57	0.09	0.04	
		Discrimination	Yes	54			61	− 0.07	0.03	
8	Diekmann et al. (1994)	Threshold	No	10			22			Lack of necessary data in the paper and the authors did not have the dataset anymore
		Discrimination	No	10			22			
		Identification	No	10			22			
9	Rosenbluth et al. (2000)	Free identification	Yes	30 (0.69)			30	0.40	0.07	
		Cued identification	Yes	30			30	0	0.07	
		Threshold	Yes	30			30	− 0.45	0.07	
10	Schwenn et al. (2002)	Threshold	Yes	14 (0.67)			14	− 0.29	0.14	
		Discrimination	Yes	14			14	0.43	0.15	
		Cued identification	Yes	14			14	0.04	0.14	
11	Zielke & Gawęcki (2003)	Threshold	Yes	50 (0.82)			50	− 0.34	0.04	
12	Wakefield et al. (2004)	Threshold	Yes	37 (0.40)	32	5	32	− 0.09	0.06	
		Free identification	Yes	37	32	5	32	0.13	0.06	
13	Hamáková (2008)	Cued identification	Yes	15 (0.20)			15	− 0.24	0.13	No separate data for cued and free identification in OMT test; no contact details of the author
		Threshold	Yes	15			15	0	0.13	
		Cued + free identification	No	15			15			
14	Cuevas et al. (2009)	Discrimination	Yes	13 (0.0)	13	0	13	1.90	0.22	
		Cued identification	Yes	13	13	0	13	0.81	0.17	
		Free identification	Yes	13	13	0	13	3.77	0.43	
15	Cuevas et al. (2010)	Threshold	Yes	8 (0.0)	8	0	16	1.73	0.25	
		Discrimination	Yes	8	8	0	16	0.95	0.21	
		Cued identification	Yes	8	8	0	16	− 0.28	0.19	
		Retronasal identification	No	8			16			Not included—retronasal olfaction
16	Rombaux et al. (2010) and Renier et al. (2013)	Discrimination	Yes	10 (0.0)	10	0	10	1.62	0.27	Two papers report results from the same group of blind people in different contexts [we included the results presented in Renier et al. (2013)]
		Free identification	Yes	10	10	0	10	3.50	0.51	
17	Kupers et al. (2011)	Threshold	Yes	11 (0.36)	11	0	14	− 0.14	0.16	Behavioral data from an fMRI study
18	Oniz et al. (2011)	Threshold	Yes	40 (0.43)			52	0.15	0.04	
		Cued identification	Yes	40			52	− 0.29	0.04	
		Discrimination	Yes	40			52	− 0.15	0.04	
19	Beaulieu-Lefebvre et al. (2011)	Threshold	Yes	11 (0.36)	11	0	14	0.78	0.17	
		Discrimination	Yes	11	11	0	14	0	0.16	
		Cued identification	Yes	11	11	0	14	0.75	0.17	
20	Luers et al. (2014)	Threshold	Yes	46 (0.33)			46	− 0.09	0.04	
		Discrimination	Yes	46			46	− 0.42	0.04	
		Cued identification	Yes	46			46	0.23	0.04	
21	Majchrzak & Eberhard (2014)	Cued identification	Yes	94 (0.53)			98	− 0.33	0.02	Average scores for people below 80 years of age obtained directly from the authors. The data were later published in a journal article (Majchrzak et al., 2017)
		Discrimination	Yes	94			98	− 0.06	0.02	
22	Gagnon et al. (2015)	Free identification	No	12			14			Data could not be included—results reported across ortho- and retronasal olfactory tasks
		Cued Identification (ortho- and retronasal testing)	No	12			14			
		Retronasal identification	No	12			14			Not included—retronasal olfaction
23	Iversen et al. (2015)	Cued identification	Yes	14 (0.50)			14	− 0.41	0.15	
24	Çomoğlu et al. (2015)	Threshold	Yes	33 (0.48)	17	16	33	2.04	0.09	
		Cued identification	Yes	33	17	16	33	0	0.06	
		Discrimination	Yes	33	17	16	33	0.73	0.06	
25	Guducu et al. (2016)	Threshold	Yes	14 (0.36)	14	0	10	0.31	0.17	
		Cued identification	Yes	14	14	0	10	− 0.15	0.17	
		Discrimination	Yes	14	14	0	10	− 0.08	0.17	
26	Sorokowska (2016)	Threshold	Yes	84 (0.54)	43	41	84	0.12	0.02	
		Cued identification	Yes	84	43	41	84	− 1.05	0.03	
		Discrimination	Yes	84	43	41	84	0.59	0.02	
		Free identification	Yes	84	43	41	84	− 0.09	0.02	
27	Cornell Kärnekull et al. (2016)	Threshold	Yes	30 (0.73)	15	15	30	− 0.22	0.07	
		Free identification	Yes	30	15	15	30	0.46	0.07	
		Discrimination	Yes	30	15	15	30	0.23	0.07	
28	Sorokowska & Karwowski (2017)	Cued identification	No	94			108			Most blind participants took part also in Sorokowska (2016) study
		Free identification	No	94			108			

For studies included in the meta-analysis we provide *g* and variance of *g* (var-*g*), and for excluded studies a reason for exclusion. Proportion of women in the blind sample and numbers of early and late-blind individuals are reported only for studies that were included in the meta-analysis and where such data were available. For studies involving more than one method of testing, the proportion of women is reported only once

### Meta-analysis inclusion criteria

The criteria for data to be included in the meta-analysis were: (1) use of a psychophysical olfactory test, (2) more than one participant in the blind or control group (“blindness” was defined following the nomenclature applied by the authors of studies included in the current meta-analysis; the samples included totally blind people, participants with light perception and legally blind individuals, i.e., people with visual acuity below 0.1), (3) data available for both blind and sighted individuals (e.g., raw data, descriptive statistics, or statistical tests for measuring group differences), (4) data only presented once (no reused data), and (5) testing orthonasal olfaction.

Some studies involved more than one method of testing (for example, free and cued odor identification tests, or full SST test that involves olfactory threshold, discrimination and identification tasks; Hummel et al., 1997). In such cases, the data reported in the study were analyzed separately for each method and subtest. Table 1 provides an overview of the included and excluded studies. For olfactory threshold, 18 studies were localized yielding a total sample of *n* = 1231 (582 blind and 649 sighted individuals), 14 studies targeted discrimination (*n* = 940: 455 blind and 485 sighted), 14 studies measured cued identification (*n* = 968: 468 blind and 500 sighted), and 9 studies (*n* = 501: 236 blind and 265 sighted individuals) assessed free identification. Overall, blind and sighted participants had a mean age of *M* = 33.72 years (SD 16.45; range of mean age across participating samples 11–75). The studies were conducted in various countries between 1909 and 2016.

### Coding procedures

The first two authors independently coded each article for relevant information, including: sample size, sample selection, main statistics necessary for the computation of effect size, and information needed for the moderator analyses (see below). Next, AS, PS and MK reviewed the coded data and articles, discussed and resolved any discrepancies to help eliminate errors in coding.

### Statistical methods

The results of our meta-analysis were estimated in three steps. First, for each of criteria of interest, i.e., threshold, discrimination, cued identification, and free identification, we conducted a standard meta-analytical approach by estimating the average effect size (Hedges *g*) using random effects meta-analysis and assessed the heterogeneity of the effects. We used Hedges *g* as a measure of effect size. In most cases (23 out of 27 studies) *g* was obtained by subtracting average results of sighted individuals from the results of blind individuals and dividing the difference by pooled standard deviation, in the remaining cases we estimated *g* based on provided results of *t* tests or ANOVA. Therefore, positive *g* (i.e., higher than 0) indicates higher scores of blind individuals, while negative *g* indicates higher scores of sighted individuals. Although *g* is known to perform better than Cohen’s *d*, it might be slightly biased in the case of small studies. Therefore, additionally we corrected all effect sizes, using a formula proposed by Hedges (Hedges & Olkin, 1985).^1^ While interpreting our findings we rely on widely accepted criteria proposed by Cohen (1977), so *g* = 0.2 was interpreted as indicating small effect, *g* = 0.5 indicating medium effect and *g* = 0.80 as indicating large effect. All effects were illustrated on forest plots demonstrating estimated weights, effect sizes and 95% confidence intervals across studies.

We assessed the heterogeneity of the effects, by applying both Cochran’s *Q* (Cochran, 1954) and *I*^2^ statistics. *Q* is a statistic useful to discriminate heterogenous effects from homogenous effects. However, the power of *Q* is too low to properly examine heterogeneity (Gavaghan et al., 2000) and it does not quantify the possible heterogeneity. Therefore, we specifically emphasize the estimates of *I*^2^, the statistic that denotes the percentage of variation across studies that may be attributed to heterogeneity (Higgins & Thompson, 2002; Higgins et al., 2003). Unlike *Q, I*^2^ is not linked with the number of studies included. According to Higgins and Thompson (2002) *I*^2^ = 25% indicates low, *I*^2^ = 50% medium and *I*^2^ = 75% high heterogeneity. We followed these benchmarks while interpreting our findings.

Second, we examined the robustness of the obtained effect sizes by examining whether they were influenced by small studies effect, or by selective publishing (i.e., publication bias). For each of our criteria, we analyzed funnel plots (Duval & Tweedie, 2000a, 2000b) and statistically estimated the possible bias using Egger’s regression test and estimating Kendall’s rank-correlation (*τ*) for funnel asymmetry. The funnel plot is a scatter plot illustrating effect sizes obtained in studies included into a meta-analysis and their precision. An effect size is put on the horizontal axis and a measure of weight (in our study a standard error, consistently with recommendations, see: Sterne & Egger, 2001) on the vertical axis. This method of analysis assumes that effect sizes observed in individual studies should be independent from their sample size, i.e., the plot should have a shape of a funnel. If smaller studies produce systematically stronger effect sizes, it might indicate an existence of some publication bias. Therefore, severe and significant asymmetry of the funnel plot might indicate that the estimates of effect sizes are not trustworthy and should be corrected. Egger’s regression and Kendall’s rank correlation quantify this asymmetry.

One of corrections is the trim-and-fill method, which “forces” a symmetrical distribution of effects around the mean, or in other words analytically “adds” additional studies (“filled studies”) required to obtain full symmetry of the plot (Duval & Tweedie, 2000a, 2000b). Although this method is widely used, its basic assumption—namely the perfect symmetry in the distribution of effects around the mean—is not very realistic (Peters, Sutton, Jones, Abrams, & Rushton, 2007). Therefore, we decided to supplement the trim-and-fill method by the so-called PET-PEESE approach (Stanley & Doucouliagos, 2014). This method fits a meta-regression model predicting effect sizes in studies by their variances (the precision effect test, called PET), or their standard errors (the precision effect estimate with standard errors, called PEESE). If the intercept is statistically significant in the PET model, the PEESE model should be taken into account as the publication-bias-free effect size. Although not without its problems (Stanley, 2017), this method seems effective in providing corrected estimates for small-studies meta-analyses (see, e.g., Carter & McCullough, 2014).

Finally, the third step of our analysis comprised an exploratory analysis targeting the role played by three *moderators of interests*: the average age of participants in included studies, the proportion of women versus men in each of the studies and onset of blindness (defined as early blind vs. late-blind participants). The moderator analysis enabled us to explore the possible role of different factors in an effort to explain the high heterogeneity of the reported effects. “Early blindness” and “late blindness” were defined following the nomenclature applied by the authors of studies included in the current meta-analysis. “Early blindness” was understood as a congenital blindness, or a complete loss of sight before the age of 2 years, i.e., before completion of visual development (Wiesel, 1982), and “late blindness” as a loss of sight after the age of 2 years. Age and proportion of women were analyzed by means of meta-regression—we included them as predictors of the effect size and examined whether any of them modified the obtained effect size. In the case of analyses with potential publication bias, the meta-regression was conducted with a PET-PEESE correction.

For the onset of blindness, our procedure differed and was conducted in 4 steps. First, across all studies that reported separate within-study results for early and late blind samples (e.g., Wakefield et al., 2004), we calculated a separate effect sizes of the difference between early blind participants versus sighted controls and late-blind participants versus sighted controls. In the second step, we created an additional database on an effect-level. In the third step, we excluded all studies where no details about onset of blindness were provided or no separate results were provided for a mixed sample of early and late-blind individuals. In the last, fourth step, we re-estimated the average effect of difference between early blind individuals vs. controls and late-blind individuals vs. controls.

All analyses were in the R statistical environment, using the metafor package (Viechtbauer, 2010).

## Results

### Threshold

**Fig. 1:**
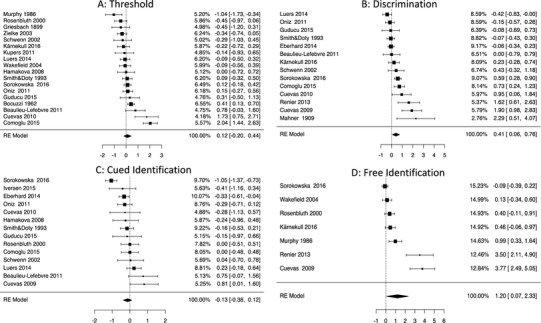
Forest plots demonstrating estimated weights, effect sizes and 95% confidence intervals across studies

**Fig. 2:**
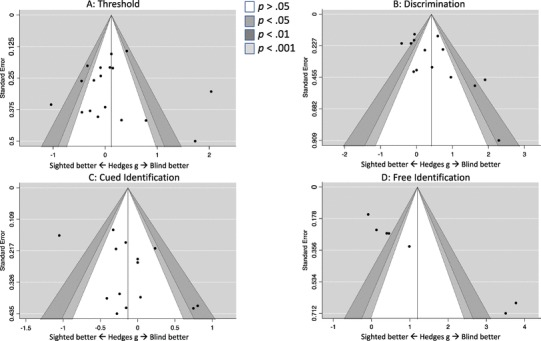
Funnel plots assessing possibility of publication bias

For odor thresholds, the obtained effects were heterogeneous, *Q*(*df* = 17) = 87.34; *p* <0 .001, thus indicating that there was a significant variability in obtained effect sizes across studies. The *I*^2^ for odor threshold was estimated at *I*^2^ = 86%, thus indicating large heterogeneity (Higgins et al., 2003). Effect size obtained in a random-effects meta-analysis indicated lack of differences between the blind and sighted individuals: *g* = 0.107; 95% CI − 0.218 to 0.433 (*p* = 0.519) (see Fig. 1a). The funnel plot was symmetrical and neither Egger’s test nor rank-correlation coefficient suggested publication bias/influence of small studies (Fig. 2a).

### Discrimination

The effect size obtained for discrimination was highly heterogeneous, *Q*(*df* = 13) = 56.79; *p* < 0.001; *I*^2^ = 83%, and the effect size obtained in random-effect meta-analysis indicated a significant low-to-moderate effect size: *g* = 0.413, 95% CI 0.064–0.763, suggesting higher discriminative skills of blind individuals (Fig. 1b). An inspection of the funnel plot (*τ* = 0.407; *p* = 0.047), however, clearly demonstrated that three small studies yielded especially large effects (*g* = 1.62–2.29) (Fig. 2b). As clarified above, we thus estimated the corrected effect using two methods—trim-and-fill method and PET-PEESE approach (both methods independently provide bias-corrected effect sizes, although they base on different assumptions). The effect size corrected for small-studies influence did not demonstrate any significant differences between blind and sighted individuals. In the case of funnel-plot-filled studies the effect was estimated at *g* = 0.11, 95% CI − 0.165 to 0.387, while in the case of PET-PEESE it was *g* = − 0.31, 95% CI − 0.82 to 0.20. Hence, the difference between blind and sighted participants disappeared when estimated with the control of possible influences of underpowered studies.

### Cued identification

The effect size obtained in the case of cued identification was also significantly and moderately heterogeneous, *Q*(*df* = 13) = 44.81; *p* < 0.001; *I*^2^ = 68.5%. The obtained results showed that the effect was not significantly different from 0: *g* = − 0.131; 95% CI − 0.378 to 0.116 (Fig. 1c). Kendall’s *τ* and Egger’s test did not suggest publication bias (see Fig. 2c).

### Free identification

Finally, the analysis of free identification showed that the obtained effect size was both significant, *Q*(*df* = 6) = 59.68; *p* < 0.001, and highly heterogeneous (*I*^2^ = 96.5%). As demonstrated in Table 2, the obtained difference between blind and sighted individuals was significant and large in terms of the effect size: *g* = 1.20, 95% CI 0.072–2.33 (see also Fig. 1d), in favor of the blind group. A closer look at the funnel plot, however (see Fig. 2d), indicated that this large effect was primarily driven by two very small studies with enormously high effect sizes (*g* = 3.50 and *g* = 3.77, respectively). The funnel plot was asymmetric (Kendall’s *τ* = 0.91; *p* = 0.003, Egger’s *z* = 7.54, *p* < 0.001), and the bias-corrected effect was no longer significant regardless of the analytical method applied. In other words, the previously observed difference between blind and sighted individuals was driven by effects from small studies.

**Table 2:** A summary of obtained effect sizes for differences between blind and sighted individuals in threshold, discrimination, cued identification and free identification

Estimate type	Effect size (Hedges *g*)	95% CI	*p*
Threshold (*k* = 18, *N* = 1227, *N*_blind_ = 590, *N*_sighted_ = 637)			
Uncorrected estimates	0.107	− 0.218 to 0.433	0.43
Publication bias test			
Egger’s test	*z* = 0.717, *p* = 0.47		
Rank-correlation test	*τ* = 0.085, *p* = 0.65		
Discrimination (*k* = 14, *N* = 940, *N*_blind_ = 455, *N*_sighted_ = 485)			
Uncorrected estimates	0.413	0.064 to 0.763	0.021
Publication bias test			
Egger’s test	*z* = 3.282, *p* < 0.001		
Rank-correlation test	*τ* = 0.407, *p* = 0.047		
Publication-bias corrected estimates			
Trim and fill	0.111	− 0.165 to 0.387	0.43
PET-PEESE	− 0.31	− 0.82 to 0.20	0.21
Cued identification (*k* = 14, *N* = 968, *N*_blind_ = 468, *N*_sighted_ = 500)			
Uncorrected estimates	− 0.131	− 0.378 to 0.116	0.30
Publication bias test			
Egger’s test	*z* = 1.83, *p* = 0.068		
Rank-correlation test	*τ* = 0.187, *p* = 0.39		
Free Identification (*k* = 7, *N* = 443, *N*_blind_ = 224, *N*_sighted_ = 219)			
Uncorrected estimates	1.20	0.072 to 2.326	0.037
Publication bias test			
Egger’s test	*z* = 7.54, *p* < 0.001		
Rank-correlation test	*τ* = 0.91, *p* = 0.003		
Publication-bias corrected estimates			
Trim and fill	0.084	− 0.409 to 0.577	0.738
PET-PEESE	0.02	− 0.26 to 0.30	0.88

*95% CI* 95% confidence intervals, *p p* value, *k* the number of studies, *N* total number of participants, *PET-PEESE* precision-effect testing–precision-effect-estimate with standard error meta-analysis

### Moderator analysis

Although two out of four effects were visibly distorted by the effects obtained in underpowered studies and none of the corrected effects was statistically significant, all observed effects were also highly heterogeneous. As mentioned in “Statistical methods”, we aimed to explain the high heterogeneity of the reported effects by means of the moderator analysis.

The meta-regression predicting the effect of difference between blind and sighted individuals in olfactory thresholds showed no effect of the proportion of women, *B* = − 1.08, SE = 0.78, *p* = 0.16, nor average participants’ age across studies, *B* = 0.002, SE = 0.009, *p* = 0.81. Neither did these two moderators differentiate the effect of olfactory discrimination: when estimated with the PET-PEESE correction, the effect for the proportion of women was estimated at *B* = 0.48, SE = 0.92, *p* = 0.61, while the effect of participants’ age was estimated at *B* = − 0.008, SE = 0.008, *p* = 0.32. In the case of cued identification, the proportion of women (*B* = − 0.61, SE = 0.65, *p* = 0.34), and the age of participants (*B* = 0.009, SE = 0.008, *p* = 0.24) did not moderate the observed effect. Similarly, no moderation by these two factors was found in the case of free identification—when corrected using PET-PEESE estimation the effect of the proportion of women was not significant, *B* = 0.88, SE = 0.59, *p* = 0.37, similarly as the effect of participants’ age, *B* = 0.01, SE = 0.007, *p* = 0.34.

In the next step, we compared the effects of difference between early-blind and late-blind individuals and their sighted counterparts. In the case olfactory threshold, there was no significant moderation effect, *Q*_between_(*df* = 1) = 0.44, *p* = 0.51. As illustrated in Table 3, both effects were similar in their size and none was statistically significant, although early blind people tended to perform slightly better than the sighted individuals (*g* = 0.45, *p* = 0.055).

**Table 3:** Onset of blindness as a moderator of difference between blind and sighted individuals in odor threshold, discrimination, identification, and free identification

	Number of included effects	*g* (95% CI)	*p*
Threshold			
Early blind samples	10	0.45 (− 0.01, 0.91)	0.055
Late blind samples	6	0.25 (− 0.30, 0.81)	0.37
Discrimination			
Uncorrected			
Early blind samples	9	0.71 (0.22, 1.20)	0.005
Late blind samples	3	0.08 (− 0.53, 0.69)	0.80
PEESE corrected			
Early blind samples	9	− 0.04 (− 0.63, 0.54)	0.85
Late blind samples	3	− 0.76 (− 7.06, 5.53)	0.37
Cued identification			
Early blind samples	6	0.08 (− 0.34,0.51)	0.70
Late blind samples	2	− 0.18 (− 0.49, 0.14)	0.28
Free identification			
Uncorrected			
Early blind samples	5	1.64 (0.47, 2.82)	0.006
Late blind samples	3	− 0.13 (− 0.63, 0.37)	0.61
PEESE corrected			
Early blind samples	5	− 0.16 (− 1.21, 0.90)	0.67
Late blind samples	3	0.16 (-4.29, 4.61)	0.46

When we compared the effects of early blind individuals versus controls with late blind individuals versus controls with reference to olfactory discrimination, no moderation was observed, *Q*_between_(*df* = 1) = 1.82, *p* = 0.18. As presented in Table 3, there was a statistically significant, positive effect in favor of early blind individuals when compared with control groups (*g* = 0.71, *p* = 0.005), while in the case of the comparison between late blind individuals and control groups the effect was not different from 0 (*g* = 0.08, *p* = 0.80). However, given our previous findings on small studies influence on discrimination and free identification tests results, we re-estimated the moderated effects using PET-PEESE correction. When the effects were corrected, they did not differ from 0 and one from each other.

The onset of blindness did not moderate the cued identification test results, *Q*_between_(*df* = 1) = 0.43, *p* = 0.51; as presented in Table 3, both effects were similar and none was significant.

Finally, in the case of free identification, there was a marginal moderating effect of the onset of blindness, *Q*_between_(*df* = 1) = 3.43, *p* = 0.06—early blind individuals outperformed their sighted counterparts (*g* = 1.64, *p* = 0.006), while there was no difference between late blind individuals and sighted controls (*g* = 0.16, *p* = 0.61). However, given identified symptoms of publication bias, we re-estimated these effects using PET-PEESE correction. When corrected, the effects did not differ from each other and none was statistically significant (see Table 3).

## Discussion

The potential presence of olfactory compensation in blindness has interested scientists for decades. Many studies conducted since the beginnings of the twentieth century explored this topic, although findings regarding olfactory compensation have been inconclusive. The results from the present meta-analysis show that the olfactory abilities of blind and sighted people are not much different overall. No positive effects from visual impairment were observed for all aspects analyzed in the current research: odor detection threshold, olfactory discrimination, free and cued odor identification abilities. In addition, age, proportion of women and blindness onset did not moderate the observed, null findings.

Consistent with what has been suggested by experts in the area of sensory compensation (Kupers & Ptito, 2014), we found that compensatory effects in smell function are not straightforward. Notably, the obtained effect sizes for all odor functions were highly heterogeneous and the observed differences between blind and sighted individuals in single studies were mostly observed in small studies. Potential explanations for this heterogeneity are further discussed below.

Most previous studies did not show significant differences between congenital and late blind participants (e.g., Çomoğlu et al., 2015; Sorokowska, 2016). However, some small, single studies indicated that the olfactory abilities of early blind groups differed from the performance of sighted people. Early blind participants performed better than sighted people in free identification (the effects were particularly strong in Cuevas et al., 2009 and in Renier et al., 2013 and Rombaux et al., 2010 studies) and discrimination tests (Cuevas et al., 2009; Mahner, 1909; Renier et al., 2013; Rombaux et al., 2010). Additionally, in the current meta-analysis we observed a slight, albeit non-significant trend indicating that early blind participants tend to perform better than sighted people in the threshold task. The findings on early blind subjects are particularly interesting, given the existing hypotheses regarding their superior olfactory performance. Probably, the observed magnitude and direction of effects in the case of early blind people results from cerebral reorganization that could support their olfactory processing. Degree of such a reorganization could change, depending on a moment of sensory loss. Although in a study involving a mixed sample of early and late-blind people (visual acuity below 0.1), Luers et al. (2014) showed that the duration of blindness does not correlate with olfactory function (*r* between 0.01 and 0.17 for SST subtests), in Majchrzak et al. (2017), the correlations reported for olfactory discrimination and identification in a sample of blind and visually impaired people were mostly positive and significant (*r* = 0.234, *p* < 0.05 for odor discrimination and *r* between − 0.48 and 0.19 for SST identification subtest, depending on the reason of visual impairment). Nevertheless, it is possible that complete loss of sight before visual development is a different case. The functional reorganization in the occipital cortex (Leclerc, Saint-Amour, Lavoie, Lassonde, & Lepore, 2000) could aid some unisensory processes, which was shown, for example, for auditory skills (Gougoux, Zatorre, Lassonde, Voss, & Lepore, 2005). However, some aspects of sensory abilities and performance can be also impeded in blindness. Absence of a calibrating visual reference frame in the congenitally blind can, for example, negatively influence multisensory spatial integration between hearing and touch (Hötting, Rösler, & Röder, 2004) or ability to localize sound sources in the vertical spatial plane (Lewald, 2002; Zwiers, Van Opstal, Cruysberg, Opstal, & Cruysberg, 2001). This illustrates how blindness could underlie both enhanced and decreased sensory skills—and might explain why the overall pattern of results in the case of olfaction is not very simple. Although calibration problems seem not to be the case for the sense of smell, there might be some additional issues, like development of specific experience-based associations that differ in sighted and blind and which result in differences in olfactory processes. Further, available neural resources could be used more extensively for modalities other than the sense of smell, not allowing for development of olfactory superiority.

Nevertheless, it needs to be highlighted that our meta-analysis shows rather minimal compensatory plasticity for olfaction which is not in line with most findings on in the unisensory tactile (Van Boven, Hamilton, Kauffman, Keenan, & Pascual-Leone, 2000) and auditory domain processing (Lessard, Paré, Lepore, & Lassonde, 1998). Based on our research, some new hypotheses might be presented as to why blind individuals do not develop very high olfactory capacities in some domains to compensate for their lack of vision.

First, the aim of sensory compensation processes is to alleviate the incapacitating consequences of sensory deficit or loss (Bäckman & Dixon, 1992). Both blind and sighted people could be equally proficient in some skills, and in this case it would be not possible to develop some olfactory abilities any better (for example, studies on cued olfactory identification typically demonstrate a strong ceiling effect; Hummel, Kobal, Gudziol, & Mackay-Sim, 2007). Second, compensatory processes could be more pronounced for other sensory modalities because the olfactory and visual data are not necessarily redundant—for example, for assessments of attractiveness, visual and olfactory cues are not consistent (Sorokowska, 2013); lack of vision would not necessarily enhance contradictory or complementary signals. Third, it is still possible that superior abilities of the blind people would be observed during processing of olfactory information outside laboratory context, in more ecologically valid studies. For example, blind people could be compared to the sighted in detection of odors in an environment containing also other smells, or in recognition of smells they would be exposed to on the way to the testing facility. Such studies would be more appropriate to test the hypothesis on the increase in olfactory performance due to more extensive, daily olfactory training of blind people and higher olfactory awareness. Further, in natural experiments, sighted people would probably not be equally focused on olfactory stimuli like during laboratory testing (e.g., in many analyzed studies the eyes of sighted people were closed or covered, thus limiting the regular sensory input). Perhaps, the olfactory superiority of the blind people would only be observed in conditions where participants’ attention would not be specifically driven to olfactory processing. Finally, in our meta-analysis, the studies in which significant differences between sighted and blind people were observed were mostly based on few observations. This might suggest a large individual variation, especially among the early blind where olfactory expertise may result from a more active attention towards olfactory information that can ultimately yield a keener sense of smell. Similar to trained subjects (employed by the Philadelphia Water Department), blind people could become better in odor detection (Smith et al., 1993), or other olfactory abilities. Such acquisition of olfactory function has also been noticed in a number of studies on “olfactory training” which suggest that olfactory function can be improved by regular, short-term exposure to odors (Sorokowska, Drechsler, Karwowski, & Hummel, 2017). However, it also needs to be noted that in studies observing the highest differences between blind and sighted, olfactory abilities in sighted were relatively low. In future studies, a more balanced sample selection is required to conduct reliable comparisons.

Another interesting issue are the effect differences across the four different olfactory tasks. For example, the highest number of negative effects (indicating slight advantage of sighted subjects over the blind participants) was observed for cued identification. As discussed in the introduction, cultural context is important for the identification test execution (e.g., Oleszkiewicz et al., 2016; Sorokowska & Hummel, 2014). It is possible that due to different way of life, blind people are exposed to different odors than the sighted people, and thus some tests which are theoretically based on common smells can be more difficult for the blind people than for the sighted. Further, the meta-analysis indicated a non-significant difference between blind and sighted individuals in olfactory threshold, although we observed a slight tendency for early blind individuals to perform better than the sighted people in this subtest, which was discussed earlier in this section. The effect in the threshold task was not moderated by publication bias or influenced by small studies. Importantly, ceiling effect is not very often observed in olfactory threshold studies, and therefore, changes in this olfactory function can be seen relatively easily. It is also crucial that threshold tests are rather independent from verbal abilities (Hedner et al., 2010; Sorokowska, Sorokowski, Hummel, & Huanca, 2013). The absent difference between sighted and blind individuals suggests that one of potential moderators of the effects observed in single studies testing other olfactory abilities could be verbal skills.

In contrast, the higher olfactory discrimination skills among blind relative sighted were driven by three small studies that yielded especially large effects. The effect disappeared when estimated with the control of possible influences of underpowered studies. Nevertheless, it needs to be remembered that in the case of olfactory discrimination, the results highly depend on the way the odors are presented (Weierstall & Pause, 2012). The outlier studies observed on our funnel plot used the same methodology, i.e., discriminating between two odors (Cuevas et al., 2009; Mahner, 1909; Renier et al., 2013; Rombaux et al., 2010), unlike, e.g., Sniffin’ Sticks, where a participant needs to identify which of the three odors is different (for details of the method see: Hummel, Kobal, Gudziol, & Mackay-Sim, 2007). This finding warrants further investigations.

Overall, blind individuals achieved 1.20 standard deviation higher scores in free identification. However, the large effect was primarily driven by two very small groups of blind people for whom enormously high effect sizes were observed; when the bias was corrected for, the effect proved unreliable. It needs to be noted that two groups participating in these studies (first in Cuevas et al., 2009; and second in Renier et al., 2013 and Rombaux et al., 2010 studies) were asked to identify the same set of 30 odorants. It is possible that for some reasons, these odorants were easier to recognize for blind people relative to the sighted. Further, higher scores in free olfactory identification might be associated with better memory and retrieval of smell descriptors in the group of early blind subjects. Although recent studies (Cornell Kärnekull et al., 2016; Sorokowska & Karwowski, 2017) showed that olfactory memory in the blind people is not better than this of sighted individuals, the retrieval of certain odor labels might be more effective among blind people, facilitating free identification of odorants. For example, Gagnon et al. (2015) showed that blind people were faster in recognizing orthonasally presented odors which could indicate that this task was easier for them than for the sighted subjects.

One interesting finding of this meta-analysis was that age did not moderate the observed effects. This is surprising, as age in general is an important factor when evaluating olfactory abilities. (Larsson, Finkel, & Pedersen, 2000; Sorokowska, Schriever, et al., 2015). However, detrimental effects of aging on the sense of smell could be partially due to an age-related decrease in cognitive abilities; that may affect some olfactory abilities (Hedner et al., 2010). If the olfactory superiority of blind people indeed results from daily smell training (Gagnon et al., 2015), this training might alleviate some of the aging effects. This is a topic that needs further investigation in future work.

Based on the review of studies presented in the introduction, we might suggest various future directions for research on olfactory performance of the blind people. First, an interesting factor that could influence performance of blind and sighted people regards the individual differences. Many studies on visual impairment have shown that some factors related to blind individuals’ daily life (e.g., physical activity) might influence several properties of perception (e.g., Seemungal, Glasauer, Gresty, & Bronstein, 2007). Likewise, it could be interesting to analyze individual differences related to olfactory perception, e.g., attention paid to olfactory stimuli, also directly in relation to olfactory functions, as these characteristics might moderate possible olfactory superiority. Second, future studies could test some elements not addressed in our general review and meta-analysis, like the odorants applied in previous research—for example, certain olfactory abilities depend also on trigeminal qualities of applied substances (Kleemann et al., 2009). However, no effect of blindness was observed in Boccuzzi (1962) for vanillin—one of a few odorants that do not produce trigeminal sensations (Doty et al., 1978). Further, as discussed in the sections on olfactory identification test results, certain adaptations of identification tasks might be necessary, so that these tests would be equally difficult for both participating groups. Third, it would be very interesting to study performance of blind people in more complex olfactory tasks, e.g., in spatial orientation (e.g., Welge-Lussen, Looser, Westermann, & Hummel, 2014), or changes-detection tasks (e.g., Croy, Krone, Walker, & Hummel, 2015). Finally, a more detailed analysis of aspects related to the performance of the late-blind groups (including effects of various reasons of visual impairment, or relationship of olfactory abilities and age of blindness onset / blindness duration like in Majchrzak et al., 2017) would be a very interesting idea for future meta-analytic studies.

A certain limitation to our study was the dependence on secondary sources for the analyzed data. Authors use different definitions of blindness, and the works analyzed in the current review and meta-analysis (all theoretically on blindness and olfaction) sometimes referred to “legally blind”, “early blind” or “congenitally blind” subjects. We recommend that future works on olfaction and blindness use more detailed and specific definitions. Other guidelines for future research on the topic of visual deprivation and olfaction include thorough analysis and control of the blindness status (late vs. congenital vs. early blind participants), and attention paid to participants with residual vision whose performance might differ from that of completely blind participants.

## Conclusion

The results indicate that overall, blind people do not have superior olfactory abilities than sighted. However, it needs to be noted that the results of single studies included in the current meta-analysis were highly heterogenous. This pattern of findings suggests that the effect of blindness on olfactory functions is not straightforward, but depends also on factors other than visual impairment itself. However, the restricted number of studies that control for such factors makes future investigations highly warranted.

## Electronic supplementary material

Below is the link to the electronic supplementary material.

Supplementary material 1 (DOCX 39 KB)

## Footnotes

1 While regular *g* is usually calculated using a formula $g=\frac{{M1 - M2}}{{{\text{SD pooled}}}},$ our formula used the correction for small sample size: $g=\frac{{M1 - M2}}{{{\text{SD pooled}}}} \times \frac{{(N - 3)}}{{(N - 2.25)}} \times \sqrt {\frac{{N - 2}}{N}} .$
