# Supplementary material for: Olfactory perception and blindness: a systematic review and meta-analysis
Source: Psychol Res. 2018 Jun 12;83(8):1595–611. doi: 10.1007/s00426-018-1035-2 (PMC6794238; doi:10.1007/s00426-018-1035-2)
Supplement: Supplementary file 1 — Supplementary material 1 (DOCX 39 KB) [file 426_2018_1035_MOESM1_ESM.docx]

**SUPPLEMENTARY FILE S1**

Description of standardized smell-testing methods presented in the “Olfaction and blindness: a systematic review and meta-analysis” – (for a more detailed overview on olfactory tests please see: Doty, 2007; Thomas Hummel, Hummel, & Welge-Lüssen, 2013; Walliczek, Negoias, Hähner, & Hummel, 2016).

1. Munich Olfaction Test – sensitivity, discrimination, and identification subtests
2. Sniffin’ Sticks Test – threshold, discrimination and identification subtests
3. UPSIT test
4. Monex 40 Sniffin’ Sticks battery
5. Retronasal identification test
6. **Munich Olfaction Test (MOT)**

In Munich Olfaction Test (Kruggel, 1989) odors are presented in 250 mL polyethylene squeeze bottles with a flip-up spout equipped with an exchangeable handmade Teflon nosepiece that fits into a subject's nostril, allowing testing of each nostril separately. The test consists of detection threshold (sensitivity) task, (quality) discrimination task, and identification and rating task.

In the sensitivity task, 6 concentrations of Phenylethanol (PEA) and Dimethyldisulfid are presented to a participant, and the task is to choose between 3 bottles in which 2 contain water and one contains the odorous solution. Similar to the Snifin’ Sticks threshold test (see below), thresholds are measured using an ascending triple-forced choice procedure, starting with the lowest concentration. Threshold is defined as the weakest concentration of a certain solution for which the subject is able to select the odor-containing bottle correctly on two consecutive trials.

In the discrimination task, eight triplets of bottles are presented in random order, with two containing the same odor and the third a different one. Subjects have to determine which of the three bottles smells differently.

In the identification task, the participants are asked to identify a correct verbal descriptor for eight commonly known odorants. In addition, the subjects are asked to rate familiarity, pleasantness, edibility, and intensity of each smell.

1. **Sniffin’ Sticks Test (SST)**

The Sniffin Sticks Test (Hummel, Sekinger, Wolf, Pauli, & Kobal, 1997; Hummel, Kobal, Gudziol, & Mackay-Sim, 2007; Kobal et al., 1996) consists three subtests – threshold, discrimination and identification. A maximum of 16 points can be obtained in each subtest. The scores of three subtests can be analyzed separately, or as a summarized Threshold-Discrimination-Identification score (TDI) – composite ‘TDI’ scores are used in diagnosis. All odors are presented in special, odor-filled pen-shaped dispensers.

In the threshold task, subjects are presented with 16 concentrations of Phenylethanol (PEA) or n-Butanol. Ascending, three-alternate forced choice (3-AFC) paradigm is employed. In the discrimination task, subjects are presented with 16 odorous triplets, with two sticks having the same odor and one smelling differently. Similar to the MOT test, participants are asked to identify the odd-smelling stick. Odor identification is based on 16 commonly known odorants is tested using 4-AFC with written/visual cues. This assessment tool is reliable, and has been validated in different countries (e.g., Oleszkiewicz et al., 2016). The odor identification part can be used without clinical assistance (Mueller et al., 2006); and an extended version of this task is available (Haehner et al., 2009; Sorokowska, Albrecht, Haehner, & Hummel, 2015).

1. **UPSIT test**

The ‘University of Pennsylvania Smell Identification Test’ (UPSIT) is a reliable, standardized odor identification test, which has been adapted and validated for use in a number of different countries (Altundag et al., 2015; Doty, Shaman, Kimmelman, & Dann, 1984; Picillo et al., 2014). It is based on the identification of odors from a list of 4 descriptors each. Original version of the UPSIT test comprises 40 odors. It also comes in various downscaled versions including a 4-item test or a 12-item test. Administration of the UPSIT does not require supervision.

1. **Monex 40 Sniffin’ Sticks battery (MONEX-40)**

Monex 40 Sniffin’ Sticks battery (Freiherr et al., 2012) is an identification test that consists of 40 different odorants. Out of the 40 items, 16 are taken from the SST identification subtest (Hummel et al., 2007). The odors are presented with four descriptors in a cued, forced-choice testing paradigm, and the subject is instructed to identify the descriptor corresponding to the presented odor.

1. **Retronasal identification test**

Retronasal smell test designed by Heilmann and collaborators (2002) consists of grocery store condiments and food items available in powder form. The substances are applied using squeezable plastic vials with a 6-cm long spout. Subjects are free to sample as much stimulant as needed for identification. In a typical trial, through the wide-opened mouth, the experimenter places approximately 0.05 g on the middle of the tongue inside the oral cavity. Each substance is identified by means of a closed set with 4 verbal items using a forced-choice procedure. There are also other retronasal olfactory tests available which are, for example, based on candy-like odor dispensers (Renner et al., 2009).

References

Altundag, A., Tekeli, H., Salihoglu, M., Cayonu, M., Yasar, H., Kendirli, M. T., & Saglam, O. (2015). Cross-culturally modified University of Pennsylvania smell identification test for a Turkish population. *American Journal of Rhinology and Allergy*, *29*(5), e138–e141. http://doi.org/10.2500/ajra.2015.29.4212

Doty, R. L. (2007). Office procedures for quantitative assessment of olfactory function. *American Journal of Rhinology*, *21*(4), 460–473. http://doi.org/10.2500/ajr.2007.21.3043

Doty, R. L., Shaman, P., Kimmelman, C. P., & Dann, M. S. (1984, February). University of Pennsylvania Smell Identification Test: a rapid quantitative olfactory function test for the clinic. *The Laryngoscope*. John Wiley & Sons, Inc. http://doi.org/10.1288/00005537-198402000-00004

Freiherr, J., Gordon, A. R., Alden, E. C., Ponting, A. L., Hernandez, M. F., Boesveldt, S., & Lundström, J. N. (2012). The 40-item Monell Extended Sniffin’ Sticks Identification Test (MONEX-40). *Journal of Neuroscience Methods*, *205*(1), 10–16. http://doi.org/10.1016/j.jneumeth.2011.12.004

Haehner, A., Mayer, A.-M., Landis, B. N., Pournaras, I., Lill, K., Gudziol, V., & Hummel, T. (2009). High test-retest reliability of the extended version of the “Sniffin’ Sticks” test. *Chemical Senses*, *34*(8), 705–11. http://doi.org/10.1093/chemse/bjp057

Heilmann, S., Strehle, G., Rosenheim, K., Damm, M., & Hummel, T. (2002). Clinical Assessment of Retronasal Olfactory Function. *Archives of Otolaryngology–Head & Neck Surgery*, *128*(4), 414–418. http://doi.org/10.1001/archotol.128.4.414

Hummel, T., Hummel, C., & Welge-Lüssen, A. (2013). Assessment of Olfaction and Gustation. In A. Welge-Luessen & T. Hummel (Eds.), *Management of smell and taste disorders: a practical guide for clinicians* (pp. 58–75). Stuttgart: Thieme. Retrieved from https://scholar.google.se/scholar?hl=en&q=Management+of+Smell+and+Taste+Disorders%3A+A+Practical+Guide+for+Clinicians.&btnG=&as_sdt=1%2C5&as_sdtp=#0

Hummel, T., Kobal, G., Gudziol, H., & Mackay-Sim, A. (2007). Normative data for the “Sniffin’ Sticks” including tests of odor identification, odor discrimination, and olfactory thresholds: an upgrade based on a group of more than 3,000 subjects. *European Archives of Oto-Rhino-Laryngology*, *264*(3), 237–43. http://doi.org/10.1007/s00405-006-0173-0

Hummel, T., Sekinger, B., Wolf, S. R., Pauli, E., & Kobal, G. (1997). “Sniffin” Sticks’: Olfactory Performance Assessed by the Combined Testing of Odour Identification, Odor Discrimination and Olfactory Threshold. *Chemical Senses*, *22*(1), 39–52. http://doi.org/10.1093/chemse/22.1.39

Kobal, G., Hummel, T., Sekinger, B., Barz, S., Roscher, S., & Wolf, S. (1996). “Sniffin’ sticks”: screening of olfactory performance. *Rhinology*, *34*(4), 222–6.

Kruggel, F. (1989). *Die Untersuchung des olfaktorischen Systems bei Patienten mit fokalen Hirnschädigungen.* Ludwig-Maximilians-Universität, München.

Mueller, C. A., Grassinger, E., Naka, A., Temmel, A. F. P., Hummel, T., & Kobal, G. (2006). A self-administered odor identification test procedure using the “Sniffin’ Sticks.” *Chemical Senses*, *31*(6), 595–598. http://doi.org/10.1093/chemse/bjj064

Oleszkiewicz, A., Taut, M., Sorokowska, A., Radwan, A., Kamel, R., & Hummel, T. (2016). Development of the Arabic version of the “Sniffin’ Sticks” odor identification test. *European Archives of Oto-Rhino-Laryngology*, *273*(5), 1179–1184. http://doi.org/10.1007/s00405-015-3718-2

Picillo, M., Pellecchia, M. T., Erro, R., Amboni, M., Vitale, C., Iavarone, A., … Barone, P. (2014). The use of University of Pennsylvania Smell Identification Test in the diagnosis of Parkinson’s disease in Italy. *Neurological Sciences : Official Journal of the Italian Neurological Society and of the Italian Society of Clinical Neurophysiology*, *35*(3), 379–83. http://doi.org/10.1007/s10072-013-1522-6

Renner, B., Mueller, C. A., Dreier, J., Faulhaber, S., Rascher, W., & Kobal, G. (2009). The Candy smell test: A new test for retronasal olfactory performance. *Laryngoscope*, *119*(3), 487–495. http://doi.org/10.1002/lary.20123

Sorokowska, A., Albrecht, E., Haehner, A., & Hummel, T. (2015). Extended version of the “Sniffin’ Sticks” identification test: test-retest reliability and validity. *Journal of Neuroscience Methods*, *243*, 111–4. http://doi.org/10.1016/j.jneumeth.2015.01.034

Walliczek, U., Negoias, S., Hähner, A., & Hummel, T. (2016). Assessment of chemosensory function using “Sniffin’ Sticks”, taste strips, taste sprays, and retronasal olfactory tests. *Current Pharmaceutical Design*, *22*(15), 2245–2252. http://doi.org/10.2174/1381612822666160216150625
